# The Influences of Process Annealing Temperature on Microstructure and Mechanical Properties of near β High Strength Titanium Alloy Sheet

**DOI:** 10.3390/ma12091478

**Published:** 2019-05-07

**Authors:** Zhaoxin Du, Yan Ma, Fei Liu, Ning Xu, Yanfei Chen, Xiaopeng Wang, Yuyong Chen, Tianhao Gong, Dong Xu

**Affiliations:** 1 School of Materials Science and Engineering, Inner Mongolia University of Technology, Hohhot 010051, China; duzhaoxin@163.com (Z.D.); 5974464mayan@163.com (Y.M.); xuningustb@163.com (N.X.); gth@imut.edu.cn (T.G.); 2 Lab of Light-Weight Novel Materials, Inner Mongolia Metallic Materials Research Institute; Ningbo 315100, China; yeffchen@163.com (Y.C.); xudong5175@163.com (D.X.); 3 National Key Laboratory of Science and Technology on Precision Heat Processing of Metals, Harbin Institute of Technology, Harbin 150001, China; wangxiaopeng@hit.edu.cn (X.W.); yychen@hit.edu.cn (Y.C.)

**Keywords:** cold rolling process, near β titanium alloy, microstructure, mechanical properties

## Abstract

The influences of process annealing temperature during cold rolling on microstructure and mechanical properties of Ti-3.5Al-5Mo-6V-3Cr-2Sn-0.5Fe near β high strength titanium alloy sheets have been investigated. Results showed that the alloy mainly included the deformation induced dislocation structures after cold rolling but no obvious band structure, twin crystal or martensite were observed in this work. The texture components, which were affected by process annealing, are mainly γ-fiber, α-fiber and weak Goss texture. The γ-fiber of alloy when process annealed at 780 °C (α/β phase field) is stronger than at 830 °C (β phase field), where the Goss texture of alloy with process annealing temperature of 830 °C is more obvious. Results of annealing heat treatments showed that the recrystallization of the cold rolled was basically completed in a relatively short time of 2 min at 750 °C for 2 min. The refinement of grain size led to a significant increase of plasticity compared to rolled alloy. Results of tensile testing of aged alloy display the excellent combination of strength and plasticity, and the cold rolled alloy with process annealed at α/β phase field exhibits the better mechanical properties than at β phase field.

## 1. Introduction

Near β titanium alloy is an important part of high strength titanium alloy, since it has many outstanding properties, such as great yield and tensile strength, excellent corrosion resistance and fatigue crack growth resistance [1,2,3]. It is well known that the high strength titanium alloys have been paid great attention by many countries in the world. After years of development, even the metallurgical cost of titanium alloy has been reduced significantly, but the restriction for application of β titanium alloys is that potential of the higher matching of mechanical properties is not fully developed and the difficulty in controlling the forming process [4]. 

Cold working is one of the efficient methods to improve the mechanical performances of metal materials. Fine grain structure can be obtained after cold working and followed by a short time annealing [5,6]. In actual engineering manufacturing production, high strength titanium alloy sheet is an important form of application, and it can be used as a matrix of composite materials [7]. In order to obtain the ideal thickness, the multi-pass cold rolling process is sometimes required due to the large deformation sheet. Therefore, the process annealing (PA) must be taken during multi-pass cold rolling process. However, researches were mainly focused on the deformation mechanisms, such as stress induced twinning and martensitic transformation of near β titanium alloys during cold rolling [8,9]. To the best of our knowledge, few researches have been studied on the influences of process annealing between cold rolling passes. 

Ti-3.5Al-5Mo-6V-3Cr-2Sn-0.5Fe alloy is a novel near β titanium alloy with great mechanical performances [10,11,12]. In this research, the effects of the process annealing during cold rolling and the subsequent thermal treatment on its microstructure and mechanical properties were investigated. The purposes of this study are to optimize the cold rolling and heat treatment processes, and to provide some theoretical foundation for the engineering preparation of high strength near β titanium alloys.

## 2. Materials and Methods 

The alloy used in this study is a near β titanium alloy, which nominal composition is Ti-3.5Al-5Mo-6V-3Cr-2Sn-0.5Fe (wt.%). The alloy ingot was supported by the Northwest Institute for Non-ferrous Metal Research, and the chemical composition was listed in Table 1. The β transformation temperature of the alloy is about 800 °C–810 °C obtained by calculation method and metallographic method. Before the cold rolling, the alloy ingot was hot rolled to 3 mm at 850 °C followed by air cooling, then annealed at 850 °C for 30 min followed by air cooling to achieve all β phases grains. Then two kinds of cold rolling processes were carried out: The reduction of the two cold rolling processes are same (40% each rolling pass, two rolling process), the thickness for finish rolling is 1 mm. Between two rolling passes, the process annealing (PA) temperature is chosen at 780 °C (α/β phase field) and 830 °C (β phase field) respectively, PA time is 10 min. 

**Figure 1:**
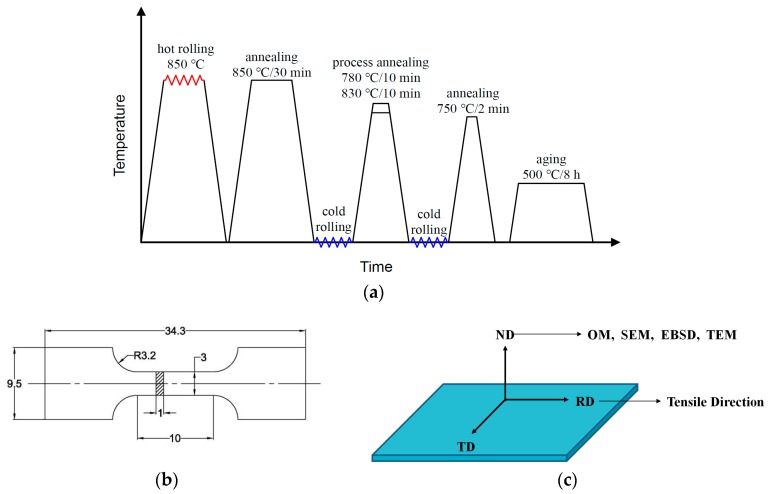
(**a**) Rolling and heat treatment procedure schematic diagram, (**b**) Dimensions of the tensile specimens (unit: mm), (**c**) Schematic diagram of sampling direction for microstructure observation and tensile testing.

The cold rolled alloys were annealed at 750 °C with the annealing time from 1 to 10 min to observe the evolution of annealing microstructure. Based on the minimum grain size which obtained during annealing, the aging heat process was carried out at 500 °C for 8 h. After annealing and aging, the samples were quickly removed from the furnace and air cooled. The rolling and heat treatment procedure schematic diagram is shown in Figure 1a.

The optical microstructures (OM) of the cold rolled and annealed alloy were obtained by Zeiss metallographic microscope. The EBSD analysis for cold rolled and annealed alloy was conducted with the step length of 3 μm on Quanta FEG 650 type field emission scanning electron microscope (FEI Company, Hillsboro, OR, USA) equipped with an Oxford Instruments Nordlys nano EBSD detector (Oxford Instruments plc, Oxfordshire, UK), and the secondary electronic scanning (SEM) image of aging microstructure was obtained by this equipment. The microstructure of cold rolled alloys was investigated by transmission electron microscopy (TEM) using FEI Tecnai G2 F20 S-TWIN (FEI Company). The samples for microstructure observation were wet polished with 400, 100, 1500 and 2000 grit silicon carbide papers and then followed by electrolytic polishing in the reagent of 60% methanol, 30% butyl alcohol, and 10% perchlorate. Samples for OM and SEM observation were etched in the solution of 3% HF, 7% HNO_3_ and 90% H_2_O.

The tensile properties of cold rolled, annealed and aged alloys were tested by uniaxial tension test method which carried out at room temperature using an Instron 5569 (Instron Company, Boston, MA, USA) machine a speed of 1 mm/min. The dimensions of the tensile samples are shown in Figure 1b. The sampling direction for microstructure observation and tensile testing as shown in Figure 1c.

## 3. Results and Discussion

### 3.1. Cold rolling and Microstructure

**Figure 2:**
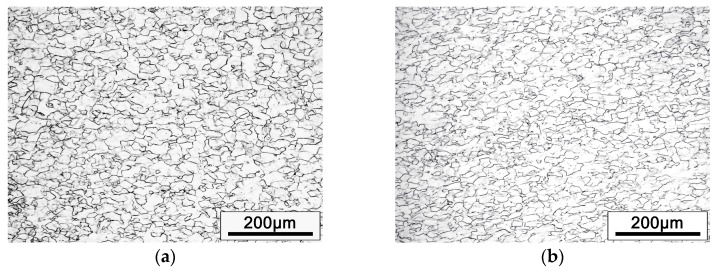
The optical microstructures of cold rolled alloys with different PA temperatures: (**a**) PA at 780 °C, (**b**) PA at 830 °C.

**Figure 3:**
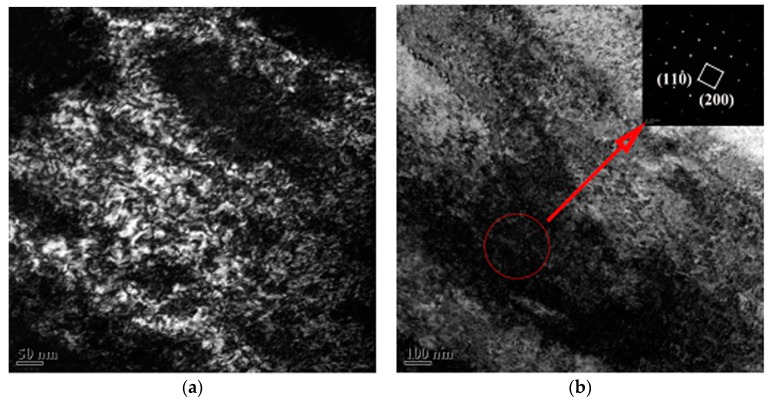
The dark field images of the cold rolled alloy PA at 780 °C: (**a**) Dark field image, (**b**) dark field image and corresponding selected area diffraction pattern.

Figure 2 shows the optical microstructure of cold rolled alloy with different PA processes. Before cold rolling, the alloy was annealed at 850 °C (β phase field), no α phase was observed in cold rolled specimens. However, the optical microstructures of the alloy seem to be no noticeable difference including the grain size. In addition, no obvious band structure, twin crystal or martensite was observed in the metallographic images, which often brought by cold rolling in β titanium alloy [13,14,15,16]. Therefore, the TEM analysis was carried out on the cold rolled alloy to observe the more detailed changes of the microstructure. Figure 3 displays the TEM examination of the cold rolled alloy with PA temperature at 780 °C. It can be seen that the alloy generated a lot of dislocations after cold rolling. The selected area diffraction pattern confirms that the selected area is the single β phase. 

**Figure 4:**
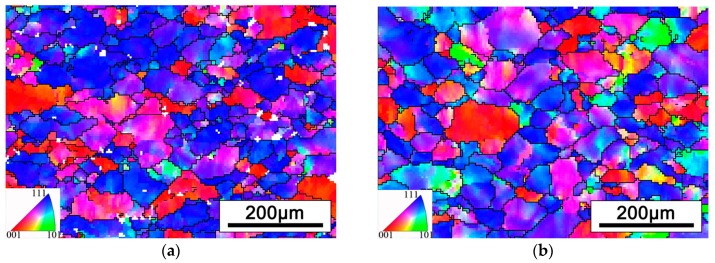
The inverse pole figure (IPF) of cold rolled alloys with different PA temperatures: (**a**) PA at 780 °C, (**b**) PA at 830 °C.

The EBSD analysis was carried out to detect more details about the microstructure of cold rolled alloy. Figure 4 displays the inverse pole figures (IPF) of the two cold rolling process alloys. The IPF maps with respect to ND axis. It can be observed that the grains of the alloys are obviously elongated along the rolling direction (RD). Moreover, red and blue are the main colors in both figures, thus, it can be deduced that there are strong textures in cold rolled alloys.

**Figure 5:**
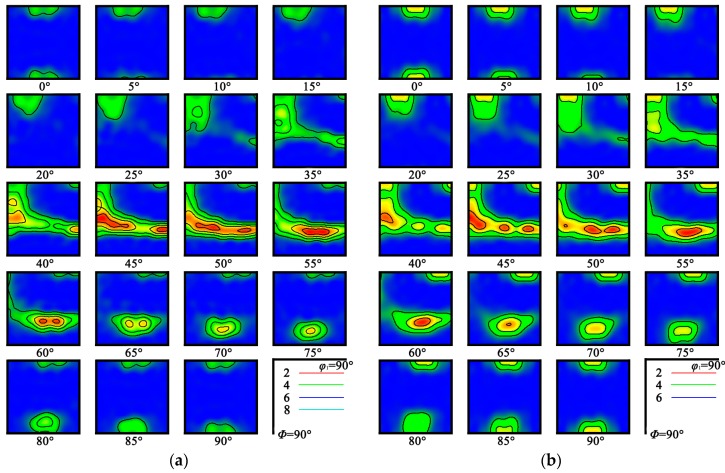
The orientation distribution function (ODF) of cold rolled alloys with different PA temperatures: (**a**) PA at 780 °C, (**b**) PA at 830 °C.

Figure 5 shows the orientation distribution function (ODF) maps of the alloy after cold rolling. In this study, the deformation mechanism of cold rolling is mainly slipped, and no twinning phenomenon has been observed. The final texture type is closely related to the slip system of the alloy. In general, {112}<111> and {011}<111> are the main slip systems in the cold deformation process of near β titanium alloys [17]. As shown in Figure 5, the cold rolled alloy exhibits strong textures, mainly including α-fiber (<110> parallel to the RD) and γ-fiber (<110> parallel to the normal direction). From observation, the cold rolled alloy has the strong γ-fiber texture components of {111}<123>, {111}<112> and {111}<110>, and α-fiber texture component of {112}<110>. In addition, the Goss texture with component of {001}<010> was also found. Very similar observations have also been carried out in previous studies [18,19]. By comparing the ODF maps of two different alloy samples, the texture of the alloy PA at α/β phase field is stronger and sharper, and mainly concentrated on the γ orientation line. However, the γ-fiber of the alloy PA at β phase field has no marked advantage over α-fiber. Also, it has a relatively obvious Goss texture, which is well known to be stabilized by the shear strain during the deformation process or by recrystallization during annealing of BCC metals [20].

### 3.2. Annealed Microstructure

**Figure 6:**
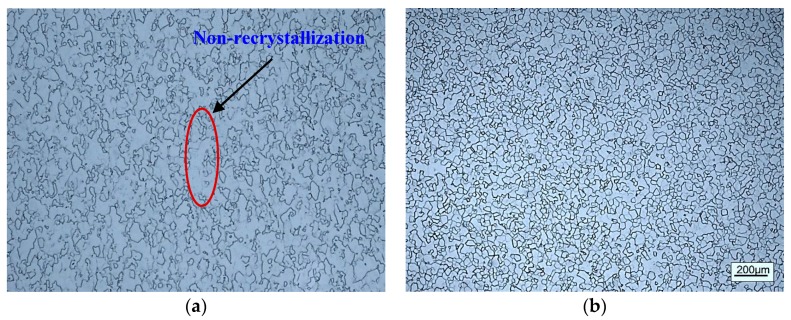

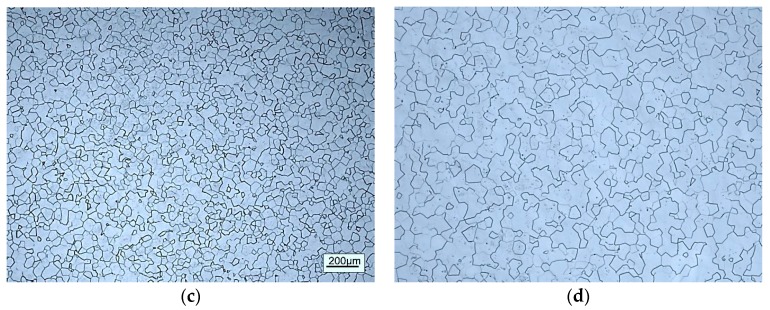
The optical microstructures of cold rolled alloy with PA temperature 780 °C annealed at 750 °C for different times: (**a**) 1 min, (**b**) 2 min, (**c**) 5 min, (**d**) 10 min.

Figure 6 displays the optical microstructures of cold rolled alloy with PA temperature 780 °C annealed at 750 °C for different times. As far as metallographic observation is concerned, there is incomplete recrystallization in the alloy annealed for 1 min as can be seen from Figure 6a. When the annealing time increased to 2 min, the rolled microstructure recrystallized and transformed into the fine equiaxed β grains (Figure 6b). Then, the β grains grown up as the annealing time continued to extend (Figure 6c,d).

The grain structure of β titanium alloy has an important influence on the mechanical properties [21]. According to the famous Hall-Petch formula [22], the strength of polycrystalline alloys always increases with the grain refinement (the increase of the total area of grain boundary). In this study, the recrystallization annealing after cold rolling can significantly refine the grain size and improve the mechanical properties of the alloy.

Generally speaking, the changes of β grain during annealing treatment include recrystallization nucleation and grain growth [23]. When the alloy undergoes large cold deformation, more energy will be stored and provide a greater driving force of recrystallization, which will lead to the faster recrystallization rate during isothermal recrystallization annealing. During the process of recrystallization nucleation, the nucleation rate of β phase can be expressed as [24]:

(1) $$I=A\;\exp\left(-\frac{Q}{RT}\right)$$

where *Q* is the nucleation activation energy, *T* is the absolute temperature and *A* depends on the composition and preparation of alloy. From Equation (1), it can be seen that the nucleation rate of β phase increases with the increase of heating temperature. In the present work, a great quantity of deformation defects such as dislocations exist in cold rolled alloys (Figure 3). Therefore, the β grain of cold rolled alloy recrystallized quickly during annealing and became coarse subsequently (Figure 6). The grain size after recrystallization has a significant effect on the mechanical properties of the alloy. The grain size could be described by [25]:

(2) $$d=n\times {\left(\frac{I}{u}\right)}^{\frac{1}{4}}$$

where *n* is a constant and u is the growth rate. From Equation (2), all factors affecting nucleation rate *I* and growth rate *u* will affect the grain size. When the amount of deformation is larger, the higher storage energies of driving nucleation and growth will be provided and then leads to the finer grain size. During the continuous heating, the β grain begins growth after recrystallization and the interfacial area and the energy of the system will be reduced [26].

**Figure 7:**
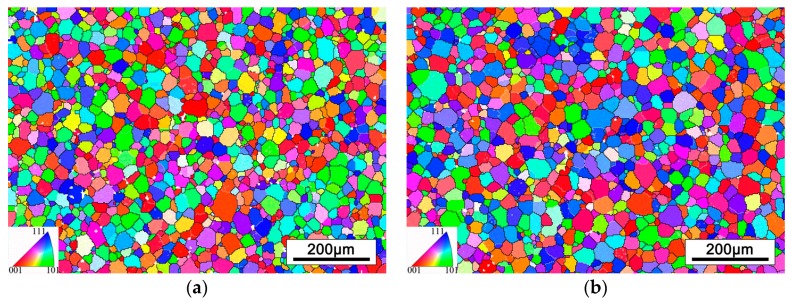
The IPF map of cold rolled annealed for 2min alloys with different PA temperatures: (**a**) PA at 780 °C, (**b**) PA at 830 °C.

Figure 7 shows the IPF map of cold rolled alloys after 2 min short time annealed. It can be seen that obvious recrystallization occurred and formed to the equiaxed β grains with fine grain size. The average grain size was measured by EBSD data, the threshold of grain minimum boundary misorientation is 15° and the minimum grain size is 6 μm. Comparing the microstructures of two kinds of rolled alloys after short time annealing, the grain sizes are 25.3 μm and 27.4 μm, respectively. It can be concluded that PA at α/β phase field is beneficial to restrain grain growth. However, there is little difference of final β grain size, due to the PA time between two rolling passes is relatively short. According to the famous Hall-Pecth equation, reducing the grain size can effectively optimize the strength of the alloy. Therefore, refining the grains by cold rolling and a short time annealing is an efficient means for improving the performances of alloy [27,28].

### 3.3. Aging Microstructure and Tensile Properties

**Figure 8:**
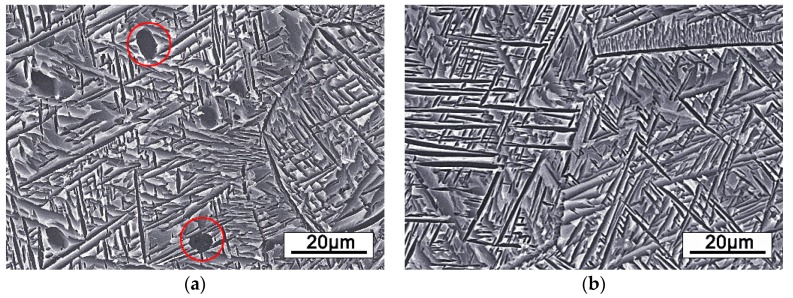
The SEM images of alloy aged at 500 °C for 8 h: (**a**) PA at 780 °C, (**b**) PA at 830 °C.

Throughout the observation of Figure 6, the alloy has the smallest grain size after 2 min annealing from the view of the optical microstructures. Therefore, the aging heat treatment was based on the microstructure after 2 min annealing. Figure 8 shows the microstructures of alloy annealed at 750 °C for 2 min and aged at 500 °C for 8 h. It can be observed that the fine needle-like secondary α phase precipitates on the β matrix during the aging treatment. As well, it can also be observed that obvious grain boundaries are used to separate α phase. Interestingly, the cold rolled alloy with PA temperature at 780 °C exist spherical α phase which is marked by red circle. Before cold rolling, the alloy was annealed at 850 °C, which indicated that a small amount of spherical α phase was produced during the PA at 780 °C between the two rolling passes. This spherical α phase is generally produced in hot deformation alloys, which is called primary α phase. Previous studies have shown that primary α phase could limit the growth of β grain [29]. Moreover, the alloy has better properties when there is bi-modal structure of primary and secondary α phases with small β grain size [10,11].

**Figure 9:**
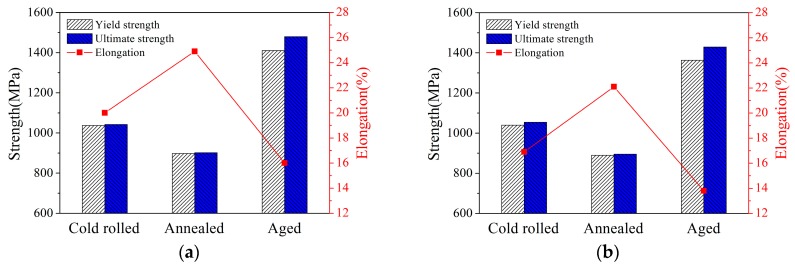
Tensile properties of alloy under different conditions: (**a**) PA at 780 °C, (**b**) PA at 830 °C.

The tensile properties of alloys under different conditions are drawn in Figure 9. It can be observed that excellent properties can be obtained by different thermal treatment processes, and the properties of alloys can be adjusted to satisfy different requirements. The alloy after cold rolling has medium strength and elongation, which can be attributed to work hardening during cold rolling. After short time annealing at 750 °C/2 min, the ductility of the alloy increases and the strength of the alloy decreases. During the subsequent aging at 500 °C/8 h, the strength of the alloy increased significantly, while the acceptable plasticity was retained. However, under the same heat treatment condition, the strength and elongation of cold rolled sheets PA at α/β phase field are higher than process annealed at β phase field. It is noteworthy that the yield strength, tensile strength and elongation of cold rolled sheets PA at α/β phase field can reach 1410 MPa, 1480 MPa and 13.8% after 750 °C/2 min plus 500 °C/8 h heat treatment. The alloy sheet has a good combination of strength and ductility by suitable heat treatment.

Secondary α phase is the most important factor affecting the properties of high strength β titanium alloys, and its precipitation is very sensitive to aging process [12]. The morphology, size and volume percentage of secondary α phase have different effects on the properties of alloy [30,31]. Fine secondary α phase forms a phenomenon similar to dispersion strengthening in the matrix of β phase [32]. The dislocation motion can be effectively restrained by the α/β interfaces. The strength of the alloy can be increased by increasing the volume fraction of the α phase and decreasing the size of the α phase. Secondary α phase can also affect the plasticity of the alloy. T. Hamajima reported that α phase is softer than β phase, leading to the deformation of α phase earlier than β phase; when the yield stress of β matrix is reached, the harder plastic deformation of small size α phase is attributed to the fast strain hardening behavior [33].

In addition, the properties of the alloy PA at α/β phase field are better than at β phase, it is mainly due to the small β grain size in PA alloy can reduce the slip length and increase crack nucleation resistance, thus improving the tensile ductility [34]. Summary, there are two main reasons for the formation of small β grain in PA alloy. First, the lower temperature provides less driving force for β grain growth during PA. Secondly, the primary α phase can be precipitated during PA at α/β phase field, which can also inhibit the growth of β grain.

## 4. Conclusions

In this study, the microstructure and mechanical properties of high strength Ti-3.5Al-5Mo-6V-3Cr-2Sn-0.5Fe alloy sheets prepared by cold rolling with different PA temperatures were investigated. The main results are summarized as follows:

(1) The alloy mainly included the deformation induced dislocation structures after cold rolling and no obvious band structure, twin crystal or martensite were observed. During the subsequent short time annealing heat treatment, the average grain size of the alloy was significantly refined.
(2) During the cold rolling process, the process annealing between each rolling pass will affect the microstructure and mechanical properties of the alloy. The alloy PA at α/β phase field shows finer grain size than at β phase field.
(3) The cold rolled alloy exhibits remarkable textures, including γ-fiber: {111}<123>, {111}<112> and {111}<110>; α-fiber {112}<110> and Goss texture {001}<010>. The γ-fiber of alloy PA at α/β phase field is stronger than that PA at β phase field, and the Goss texture of alloy PA at β phase field is more obvious.
(4) The cold rolled alloy exhibits excellent mechanical properties after short time annealing plus aging treatment. And the strength and elongation of cold rolled alloy PA at α/β phase field are both better than at β phase field due to the lower temperature and the precipitation of primary α phase.

## Figures and Tables

**Table 1:** Chemical composition of experimental alloy.

Elements	Ti	Al	Mo	V	Cr	Sn	Fe	O	N	H
**wt.%**	Bal.	3.76	4.81	6.07	2.95	2.22	0.605	0.13	0.014	0.001

## References

[1] Leyens C., Peters M. (2003). Titanium and Titanium Alloys: Fundamentals and Applications.

[2] Boyer R.R., Briggs R.D. (2005). The use of β titanium alloys in the aerospace industry. J. Mater. Eng. Perform..

[3] Cotton J.D., Briggs R.D., Boyer R.R., Tamirisakandala S., Russo P., ShchetnikovJohn N., Fanninget J.C. (2015). State of the Art in Beta Titanium Alloys for Airframe Applications. JOM.

[4] Nyakana S.L., Fanning J.C., Boyer R.R. (2005). Quick Reference Guide for β Titanium Alloys in the 00s. J. Mater. Eng. Perform..

[5] Cai M.H., Lee C.Y., Lee Y.K. (2012). Effect of grain size on tensile properties of fine-grained metastable β titanium alloys fabricated by stress-induced martensite and its reverse transformations. Scr. Mater..

[6] Cai M.H., Lee C.Y., Kang S., Lee Y.K. (2011). Fine-grained structure fabricated by strain-induced martensite and its reverse transformations in a metastable β titanium alloy. Scr. Mater..

[7] Ma Z.Y., Tjong S.C., Gen L. (2000). In-situ Ti-TiB metal-matrix composite prepared by a reactive pressing process. Scr. Mater..

[8] Min X., Emura S., Chen X., Zhou X., Tsuzaki K., Tsuchiyab K. (2016). Deformation microstructural evolution and strain hardening of differently oriented grains in twinning-induced plasticity β titanium alloy. Mater. Sci. Eng. A.

[9] Ahmed M., Wexler D., Casillas G., Savvakinc D.G., Perelomaab E.V. (2016). Strain rate dependence of deformation-induced transformation and twinning in a metastable titanium alloy. Acta Metall..

[10] Du Z.X., Xiao S.L., Xu L.J., Tian J., Kong F.T., Chen Y.Y. (2014). Effect of heat treatment on microstructure and mechanical properties of a new β high strength titanium alloy. Mater. Des..

[11] Du Z.X., Xiao S.L., Shen Y.P., Liu J.S., Liu J., Xu L.J., Kong F.T., Chen Y.Y. (2015). Effect of hot rolling and heat treatment on microstructure and tensile properties of high strength beta titanium alloy sheets. Mater. Sci. Eng. A.

[12] Chen Y.Y., Du Z.X., Xiao S.L., Xu L.J., Tian J. (2014). Effect of aging heat treatment on microstructure and tensile properties of a new β high strength titanium alloy. J. Alloys Compd..

[13] Sadeghpour S., Abbasi S.M., Morakabati M. (2015). Deformation-induced martensitic transformation in a new metastable β titanium alloy. J. Alloys Compd..

[14] Zhang J., Tasan C.C., Lai M.J., Dippel A.C., Raabe D. (2017). Complexion-mediated martensitic phase transformation in Titanium. Nat. Commun..

[15] Cho K., Niinomi M., Nakai M., Liu H.H., Santos P.F., Itoh Y., Ikeda M., Gepreel M.A.H., Narushimag T. (2016). Improvement in mechanical strength of low-cost β-type Ti–Mn alloys fabricated by metal injection molding through cold rolling. J. Alloys Compd..

[16] Sun J.F., Zhang Y., Marteleur M., Brozeka C., Rauchcd E.F., Veron M., Vermaut P., Jacques P.J., Primaa F. (2015). A new titanium alloy with a combination of high strength, high strain hardening and improved ductility. Scr. Mater..

[17] Xu Y.F., Yi D.Q., Liu H.Q., Wu X.Y., Wang B., Yang F.L. (2012). Effects of cold deformation on microstructure, texture evolution and mechanical properties of Ti-Nb-Ta-Zr-Fe alloy for biomedical applications. Mater. Sci. Eng. A.

[18] Choi G., Lee K. (2017). Effect of cold rolling on the microstructural evolution of new β-typed Ti-6Mo-6V-5Cr–3Sn-2.5 Zr alloys. Mater. Charact..

[19] Xu T.W., Li J.S., Zhang S.S., Zhang F.S., Liu X.H. (2016). Cold deformation behavior of the Ti-15Mo-3Al-2.7 Nb-0.2Si alloy and its effect on α precipitation and tensile properties in aging treatment. J. Alloys Compd..

[20] Hölscher M., Raabe D., Lücke K. (1994). Relationship between rolling textures and shear textures in fcc and bcc metals. Acta Metall. Mater..

[21] Ivasishin O.M., Markovsky P.E., Matviychuk Y.V., Semiatin S.L., Ward C.H., Fox S. (2008). A comparative study of the mechanical properties of high-strength β-titanium alloys. J. Alloys Compd..

[22] Shao C., Hui W., Zhang Y., Weng Y. (2017). Microstructure and mechanical properties of hot-rolled medium-Mn steel containing 3% aluminum. Mater. Sci. Eng. A.

[23] Ma Y., Liu J., Lei J., Liu Y., Yang R. (2009). β-grain growth and influence of its grain size on damage-tolerance property in titanium alloy. Rare Met. Mater. Eng..

[24] Li Q., Jiao X. (2019). Recrystallization mechanism and activation energies of severely-deformed magnesium during annealing process. Materialia.

[25] Hu G.X., Cai X., Rong Y.H. (2010). Fundamentals of Materials Science.

[26] Atkinson H.V. (1988). Overview no. 65: Theories of normal grain growth in pure single phase systems. Acta Metal..

[27] Li C.L., Mi X.J., Ye W.J., Hui S.X., Yu Y., Wang W.Q. (2013). A study on the microstructures and tensile properties of new beta high strength titanium alloy. J. Alloys Compd..

[28] Markovsky P.E., Bondarchuk V.I., Herasymchuk O.M. (2015). Influence of grain size, aging conditions and tension rate on the mechanical behavior of titanium low-cost metastable beta-alloy in thermally hardened condition. Mater. Sci. Eng. A.

[29] Duerig T.W., Terlinde G.T., Williams J.C. (1980). Phase transformations and tensile properties of Ti-10V-2Fe-3Al. Metall. Trans. A.

[30] Devaraj A., Joshi V.V., Srivastava A., Manandhar S., Moxson V., Duz V.A., Lavender C. (2016). A low-cost hierarchical nanostructured beta-titanium alloy with high strength. Nat. Commun..

[31] Ivasishin O.M., Markovsky P.E., Semiatin S.L., Ward C.H. (2005). Aging response of coarse-and fine-grained β titanium alloys. Mater. Sci. Eng. A.

[32] Terlinde G.T., Duerig T.W., Williams J.C. (1983). Microstructure, tensile deformation, and fracture in aged Ti 10V-2Fe-3Al. Metall. Trans. A.

[33] Hamajima T., Lütjering G., Weissmann S. (1973). Importance of slip mode for dispersion-hardened β-titanium alloys. Metall. Trans..

[34] Sauer C., Lütjering G. (2001). Influence of α layers at β grain boundaries on mechanical properties of Ti-alloys. Mater. Sci. Eng. A.

